# Extraordinary optical transmission through periodic Drude-like graphene sheets using FDTD algorithms and its unconditionally stable approximate Crank–Nicolson implementation

**DOI:** 10.1038/s41598-020-74552-5

**Published:** 2020-10-15

**Authors:** Shihong Wu, Yumei Sun, Mingmei Chi, Xiangguang Chen

**Affiliations:** 1 Department of Electrical and Electronic Engineering, College of Engineering, Yantai Nanshan University, Longkou, 265713 China; 2 School of Chemistry and Chemical Engineering, Beijing Institute of Technology, Beijing, 100081 China

**Keywords:** Engineering, Mathematics and computing, Optics and photonics

## Abstract

Based upon the approximate Crank–Nicolson (CN) finite-difference time-domain method implementation, the unconditionally stable algorithm is proposed to investigate the wave propagation and transmission through extremely thin graphene layers. More precisely, by incorporating the CN Douglas–Gunn algorithm, the piecewise linear recursive convolution method and the auxiliary differential equation method, the analytical model is proposed for Drude-like graphene model. To obtain the solution of the governing equations, the perfectly matched layer and the periodic boundary condition are applied to the graphene structure with two dimensional nano-materials. Numerical examples are carried out for further investigation. During the simulation, the influences of the parameters such as the grating slit and its thickness on the wave transmission are investigated and discussed. The result shows that not only the graphene grating has high transmission performance but also the proposed methods have considerable performance and accuracy.

## Introduction

Graphene sheet (GS), a two dimensional one-atomic-thick monolayer of graphite, is the strongest ever measured and the thinnest known material^1,2^. Graphene has attracted much attention during the last decades due to its very interesting properties, such as mechanical, physical, electrical and optical properties^3,4^. For the remarkable physical property, GSs are being studied in the field of communication and optoelectronic nanodevices^5,6^. Graphene is an ideal plasmonic material to explore tunable devices, including the tunable nanoantennas and the tunable reflectors in the range of terahertz and mid-infrared frequencies^7^. In the recent year, the absorption enhancement of graphene in near-infrared light has attracted considerable interest due to its unique electronic and optical properties^8,9^. Graphene is a kind of lossy and multi-scale material, and thus it is difficult to accurately simulate the wave propagation characteristics of GSs^10^.

The finite-difference time-domain (FDTD) method can be used as an effective numerical analysis technique to model the wave propagation in GSs because of its direct iterative solution in the time domain^11^. Shen et. al. studied the wave propagation in thin GSs by using the FDTD method^12^. Compared with a theoretical solution, they verified the proposed FDTD method through two numerical examples, and proved its accuracy and efficiency. Liang et. al. investigated the extraordinary optical transmission of periodic metallic gratings based on the locally one-dimensional FDTD^13^. In^14^, the analysis of electromagnetic phenomena in graphene structures was carried out by an efficient FDTD formulation. Numerical verification, involving comparison with closed-form solutions and the results of other schemes, exhibited the high accuracy of the algorithm.

However, analytical models and mesh sizes are key for obtaining correct results in numerical analysis technique. In order to ensure the stability of the numerical scheme, an adequate time step must be taken to assure the Courant-Friedrichs-Lewy (CFL) condition. The CFL limit is determined by the smallest cell size in the space domain^15^. However, when calculating the large amount of time steps problems, the simulation duration will be unacceptable resulting in the limitation application of the FDTD algorithm. In order to improve the computational efficiency and maintain the accuracy, the unconditionally stable algorithms are proposed. Among existed algorithms, the Crank–Nicolson (CN) algorithm with higher computational accuracy is firstly proposed in one dimensional cases^16^. By applying the original CN algorithm directly to 2D problems, large sparse matrices must be calculated at each step resulting in the computational more expensive. To avoid the calculation of the sparse matrices, approximate CN algorithms are introduced including the CN approximate-decoupling (CNAD) and CN Douglas–Gunn (CNDG) algorithms^17,18^. It has been testified that the CNDG algorithm shows higher accuracy compared with CNAD algorithm^19^. In addition, from the previously works, CNDG algorithm has been extensively employed merely in TM cases^19–21^. This limits the development of the CNDG algorithm.

In order to simulate the infinite computational domain in the limited space, an efficient absorbing boundary condition is necessary to truncate the computational domain, such as the perfectly matched layer (PML)^22^. Among modified PML schemes, unsplit-field SC-PML is developed to simplify the corners and edges of the PML regions^23^. The CFS-PML with advantages in attenuating low-frequency propagation waves and reducing late-time reflections is carried out^24^. It has been testified that the low-frequency propagation waves can not be absorbed efficiently by employing both SC-PML and CFS-PML. Thus, the higher order PML is proposed not only to alleviate such problem but also further enhanced the absorbing performance^25–27^.

Moreover, the periodic boundary condition (PBC) is also applied due to the existence of the periodic structure^15^. Among existed CN-PML algorithms, the employment of PBC have not been investigated^19–21^. Due to the existence of PBC, the Thomas algorithms can not be employed directly to the unconditionally stable algorithm. Thus, an alternative method should be investigated.

Here, based on the CNDG algorithm, higher order PML and Drude model, unconditionally stable implementation is proposed for evaluating the wave propagation characteristics through GSs in TE case. Combining with the piecewise linear recursive convolution (PLRC) and the auxiliary differential equation (ADE) methods, the extraordinary optical transmission through extremely thin periodic graphene can be simulated and calculated. Two numerical examples with different situations in one period are discussed. The results reveal the characteristics of extremely thin graphene and efficiency of the proposed algorithm.

## Theoretical approach

The Drude dispersion model can be applied to GSs^22^. According to the Drude model, the relative dielectric function $\varepsilon_{r} \left( \omega \right)$ is given by

1 $$ \varepsilon_{r} \left( \omega \right) = \varepsilon_{\infty } + \frac{{\omega_{p}^{2} }}{{ - \omega^{2} + j\omega \nu }} $$

where $\omega$ is the angular frequency of the impinging light, $\varepsilon_{\infty }$ is the relative permittivity at infinite frequency, $\nu$ is the damping constant in the graphene sheet, and $\omega_{p}$ is the plasma frequency given by

2 $$ \omega_{p} = \sqrt {\frac{{ne^{2} }}{{m^{ * } \varepsilon_{0} }}} $$

where *n* is the density of electrons, $m^{ * }$ is the electron effective mass, *e* is the electron charge, and $\varepsilon_{0}$ is the permittivity of the vacuum^23,24^. The source-free and normalized frequency-domain modified Maxwell’s curl equations in two dimensional Drude media can be written as

3 $$ j\omega \varepsilon_{0} \varepsilon \left( \omega \right)E_{x} = - \frac{{1}}{{S_{z} }}\frac{{\partial H_{y} }}{\partial z} $$

4 $$ j\omega \varepsilon_{0} \varepsilon \left( \omega \right)E_{z} = \frac{1}{{S_{x} }}\frac{{\partial H_{y} }}{\partial x} $$

5 $$ j\omega \mu_{0} H_{y} = \frac{1}{{S_{x} }}\frac{{\partial E_{z} }}{\partial x} - \frac{1}{{S_{z} }}\frac{{\partial E_{x} }}{\partial z} $$

where $S_{\eta } , \, \eta = x,z$ is the stretched coordinate variables in the higher order PML regions, given as

6 $$ S_{\eta } = \left( {\kappa_{\eta 1} + \frac{{\sigma_{\eta 1} }}{{\alpha_{\eta 1} + j\omega \varepsilon_{0} }}} \right)\left( {\kappa_{\eta 2} + \frac{{\sigma_{\eta 2} }}{{\alpha_{\eta 2} + j\omega \varepsilon_{0} }}} \right) $$

where $\kappa_{\eta n}$ is assumed to be positive integer, $\sigma_{\eta n}$ and $\alpha_{\eta n}$ are assumed to be positive, $n = 1, \, 2$. By employing the ADE approach, $S_{\eta }^{ - 1}$ can be rewritten as

7 $$ S_{\eta }^{ - 1} = k_{\eta } \frac{{j\omega + a_{\eta 1} }}{{j\omega + b_{\eta 1} }}\frac{{j\omega + a_{\eta 2} }}{{j\omega + b_{\eta 2} }} $$

where the coefficients can be given as $a_{\eta n} = {{\alpha_{\eta n} } \mathord{\left/ {\vphantom {{\alpha_{\eta n} } {\varepsilon_{0} }}} \right. \kern-\nulldelimiterspace} {\varepsilon_{0} }},b_{\eta n} = {{\alpha_{\eta n} } \mathord{\left/ {\vphantom {{\alpha_{\eta n} } {\varepsilon_{0} }}} \right. \kern-\nulldelimiterspace} {\varepsilon_{0} }} + {{\sigma_{\eta n} } \mathord{\left/ {\vphantom {{\sigma_{\eta n} } {\kappa_{\eta n} \varepsilon_{0} }}} \right. \kern-\nulldelimiterspace} {\kappa_{\eta n} \varepsilon_{0} }},k_{\eta } = {1 \mathord{\left/ {\vphantom {1 {\kappa_{\eta 1} }}} \right. \kern-\nulldelimiterspace} {\kappa_{\eta 1} }} \cdot {1 \mathord{\left/ {\vphantom {1 {\kappa_{\eta 2} }}} \right. \kern-\nulldelimiterspace} {\kappa_{\eta 2} }}$. By substituting (7) into (3)-(5), the equations can be rewritten as

8 $$ j\omega \varepsilon_{0} \varepsilon \left( \omega \right)E_{x} = - k_{z} \frac{{j\omega + a_{z1} }}{{j\omega + b_{z1} }}\frac{{j\omega + a_{z2} }}{{j\omega + b_{z2} }}\frac{{\partial H_{y} }}{\partial z} $$

9 $$ j\omega \varepsilon_{0} \varepsilon \left( \omega \right)E_{z} = k_{x} \frac{{j\omega + a_{x1} }}{{j\omega + b_{x1} }}\frac{{j\omega + a_{x2} }}{{j\omega + b_{x2} }}\frac{{\partial H_{y} }}{\partial x} $$

10 $$ j\omega \mu_{0} H_{y} = k_{x} \frac{{j\omega + a_{x1} }}{{j\omega + b_{x1} }}\frac{{j\omega + a_{x2} }}{{j\omega + b_{x2} }}\frac{{\partial E_{z} }}{\partial x} - k_{z} \frac{{j\omega + a_{z1} }}{{j\omega + b_{z1} }}\frac{{j\omega + a_{z2} }}{{j\omega + b_{z2} }}\frac{{\partial E_{x} }}{\partial z} $$

Introducing the auxiliary variables, they can be given as the following forms

11 $$ F_{\eta 1} = k_{\eta } \frac{1}{{j\omega + b_{\eta 1} }}\frac{{\partial H_{y} }}{\partial \eta } \Rightarrow j\omega F_{\eta 1} + b_{\eta 1} F_{\eta 1} = k_{\eta } \frac{{\partial H_{y} }}{\partial \eta } $$

12 $$ F_{\eta 2} = \frac{{j\omega + a_{\eta 2} }}{{j\omega + b_{\eta 2} }}F_{\eta 1} \Rightarrow j\omega F_{\eta 2} + b_{\eta 2} F_{\eta 2} = j\omega F_{\eta 1} + a_{\eta 2} F_{\eta 1} $$

13 $$ G_{\eta 1} = k_{\eta } \frac{1}{{j\omega + b_{\eta 1} }}\frac{{\partial E_{{\tilde{\eta }}} }}{\partial \eta } \Rightarrow j\omega G_{\eta 1} + b_{\eta 1} G_{\eta 1} = k_{\eta } \frac{{\partial E_{{\tilde{\eta }}} }}{\partial \eta } $$

14 $$ G_{\eta 2} = \frac{{j\omega + a_{\eta 2} }}{{j\omega + b_{\eta 2} }}G_{\eta 1} \Rightarrow j\omega G_{\eta 2} + b_{\eta 2} G_{\eta 2} = j\omega G_{\eta 1} + a_{\eta 2} G_{\eta 1} $$

where $\tilde{\eta }$ is the rest part of the field components, for example, when calculating $H_{y}$ , $\eta = x$ while $\tilde{\eta } = z$. Thus, the Maxwell’s equations in the higher order PML regions can be expressed by the auxiliary variables in the time-harmonic domain as

15 $$ \varepsilon_{0} \varepsilon \left( \omega \right)\frac{{\partial E_{x} }}{\partial t} = - \frac{{\partial F_{z2} }}{\partial t} - a_{y1} F_{z2} $$

16 $$ \varepsilon_{0} \varepsilon \left( \omega \right)\frac{{\partial E_{z} }}{\partial t} = \frac{{\partial F_{x2} }}{\partial t} + a_{x1} F_{x2} $$

17 $$ \mu_{0} \frac{{\partial H_{y} }}{\partial t} = \frac{{\partial G_{x2} }}{\partial t} + a_{x1} G_{x2} - \frac{{\partial G_{z2} }}{\partial t} - a_{z1} G_{z2} $$

By substituting (11)-(14) into (15)-(17), the equations can be rewritten as

18 $$ \varepsilon_{0} \varepsilon \left( \omega \right)\frac{{\partial E_{x} }}{\partial t} = - \left( {a_{z1} - b_{z2} } \right)F_{z2} - \left( {a_{z2} - b_{z1} } \right)F_{z1} - k_{z} \frac{{\partial H_{y} }}{\partial z} $$

19 $$ \varepsilon_{0} \varepsilon \left( \omega \right)\frac{{\partial E_{z} }}{\partial t} = \left( {a_{x1} - b_{x2} } \right)F_{x2} + \left( {a_{x2} - b_{x1} } \right)F_{x1} + k_{x} \frac{{\partial H_{y} }}{\partial x} $$

$$ \mu_{0} \frac{{\partial H_{y} }}{\partial t} = \left( {a_{x2} - b_{x1} } \right)G_{x1} + \left( {a_{x1} - b_{x2} } \right)G_{x2} + k_{x} \frac{{\partial E_{z} }}{\partial x} - $$

20 $$ \left( {a_{z2} - b_{z1} } \right)G_{z1} - \left( {a_{z1} - b_{z2} } \right)G_{z2} - k_{z} \frac{{\partial E_{x} }}{\partial z} $$

Based on the CN scheme and PLRC methods, we obtain the FDTD difference equations as follows^25,26^

21 $$ E_{x}^{n + 1} = a_{1} E_{x}^{n} + a_{2} \Psi_{ex}^{n} - p_{1z} F_{z2}^{n} - p_{2z} F_{z1}^{n} - p_{3z} \delta_{z} \left( {H_{y}^{{n + {1}}} { + }H_{y}^{n} } \right) $$

22 $$ E_{z}^{n + 1} = a_{1} E_{z}^{n} + a_{2} \Psi_{ez}^{n} + p_{1x} F_{x2}^{n} + p_{2x} F_{x1}^{n} + p_{3x} \delta_{x} \left( {H_{y}^{{n + {1}}} { + }H_{y}^{n} } \right) $$

23 $$ H_{y}^{n + 1} = H_{y}^{n} + p_{4x} G_{x2} + p_{5x} G_{x1} + p_{6x} \delta_{x} \left( {E_{z}^{n + 1} + E_{z}^{n} } \right) - p_{4z} G_{z2} - p_{5z} G_{z1} - p_{6z} \delta_{z} \left( {E_{x}^{n + 1} + E_{x}^{n} } \right) $$

where the coefficients can be expressed as

$$a_{1} = \frac{{\varepsilon_{\infty } - \xi^{0} }}{{\varepsilon_{\infty } + \chi^{0} - \xi^{0} }}$$, $$a_{2} = \frac{1}{{\varepsilon_{\infty } + \chi^{0} - \xi^{0} }}$$and

$$\chi^{0} = \frac{{\omega_{p}^{2} }}{\nu }\Delta t - \frac{{\omega_{p}^{2} }}{{\nu^{2} }}\left( {1 - e^{ - \nu \Delta t} } \right)$$, $$\xi^{0} = \frac{{\omega_{p}^{2} }}{2\nu }\Delta t - \frac{{\omega_{p}^{2} }}{{\nu^{3} \Delta t}}\left[ {1 - \left( {1 + e^{ - \nu \Delta t} } \right)e^{ - \nu \Delta t} } \right]$$

$$\Delta \chi^{0} = - \frac{{\omega_{p}^{2} }}{{\nu^{2} }}\left( {1 - e^{ - \nu \Delta t} } \right)^{2}$$,$$\Delta \xi^{0} = - \frac{{\omega_{p}^{2} }}{{\nu^{3} \Delta t}}\left[ {1 - \left( {1 + e^{ - \nu \Delta t} } \right)e^{ - \nu \Delta t} } \right]\left( {1 - e^{ - \nu \Delta t} } \right)$$and

$$ \begin{aligned} p_{1\eta } & = a_{2} \Delta t(a_{\eta 1} - b_{\eta 2} )/\varepsilon_{0} ,p_{2\eta } = a_{2} \Delta t(a_{\eta 2} - b_{\eta 1} )/\varepsilon_{0} ,p_{3\eta } = a_{2} \Delta tk_{\eta } /(2\varepsilon_{0} ) \\ p_{4\eta } & = \Delta t(a_{\eta 1} - b_{\eta 2} )/\mu_{0} ,p_{5\eta } = \Delta t(a_{\eta 2} - b_{\eta 1} )/\mu_{0} ,p_{6\eta } = \Delta tk_{\eta } /(2\mu_{0} ), \\ \end{aligned} $$

And the operator $\delta_{\eta }$ represents the CN scheme, for example,

24 $$ \delta_{z} H_{y}^{n} = \frac{{H_{y}^{n} \left( {i + 1/2,j,k + 1/2} \right) + H_{y}^{n} \left( {i + 1/2,j,k - 1/2} \right)}}{2\Delta z} $$

The updated equation of auxiliary field obtained by PLRC method can be calculated as

25 $$ \Psi_{ex}^{n + 1} = \left( {\Delta \chi^{0} - \Delta \xi^{0} } \right) \, E_{x}^{n + 1} + \Delta \xi^{0} E_{x}^{n} + e^{ - \nu \Delta t} \Psi_{ex}^{n} $$

26 $$ \Psi_{ez}^{n + 1} = \left( {\Delta \chi^{0} - \Delta \xi^{0} } \right) \, E_{z}^{n + 1} + \Delta \xi^{0} E_{z}^{n} + e^{ - \nu \Delta t} \Psi_{ez}^{n} $$

By substituting (21) and (22) into (23), the updated equation of magnetic component in the y-direction can be given as

$$ \begin{gathered} \left( {1 - D_{2x} - D_{2z} } \right)H_{y}^{n + 1} = \hfill \\ \left( {1 + D_{2x} + D_{2z} } \right)H_{y}^{n} + p_{4x} G_{x2} + p_{5x} G_{x1} - p_{4z} G_{z2} - p_{5z} \hfill \\ + p_{1x} p_{6x} \delta_{x} F_{x2}^{n} + p_{2x} p_{6x} \delta_{x} F_{x1}^{n} + p_{1z} p_{6z} \delta_{z} F_{z2}^{n} + p_{2z} p_{6z} \delta_{z} F_{z1}^{n} G_{z1} \hfill \\ \end{gathered} $$

27 $$ + \left( {1 + a_{1} } \right)p_{6x} \delta_{x} E_{z}^{n} + a_{2} p_{6x} \delta_{x} \Psi_{ez}^{n} - \left( {1 + a_{1} } \right)p_{6z} \delta_{z} E_{x}^{n} - a_{2} p_{6z} \delta_{z} \Psi_{ex}^{n} $$

where $D_{2\eta } = p_{3\eta } p_{6\eta } \delta_{\eta } \delta_{\eta }$. By employing the CNDG algorithm, adding $D_{2x} D_{2z} H_{y}^{n + 1}$ and $D_{2x} D_{2z} H_{y}^{n}$ at both sides of the equations and split the resultants, one obtains

28 $$ \left( {1 - D_{2x} } \right)\left( {1 - D_{2z} } \right)H_{y}^{n + 1} = \left( {1 + D_{2x} + D_{2z} + D_{2x} D_{2z} } \right)H_{y}^{n} + {\mathbf{A}}^{n} $$

where ${\mathbf{A}}^{n}$ is the other terms at right sides of (27). The equations can be solved by two steps as

29 $$ \left( {1 - D_{2x} } \right)H_{y}^{*} = \left( {1 + D_{2x} + 2D_{2z} } \right)H_{y}^{n} + {\mathbf{A}}^{n} $$

30 $$ \left( {1 - D_{2z} } \right)H_{y}^{n + 1} = H_{y}^{*} - D_{2z} H_{y}^{n} $$

**Figure 1:**
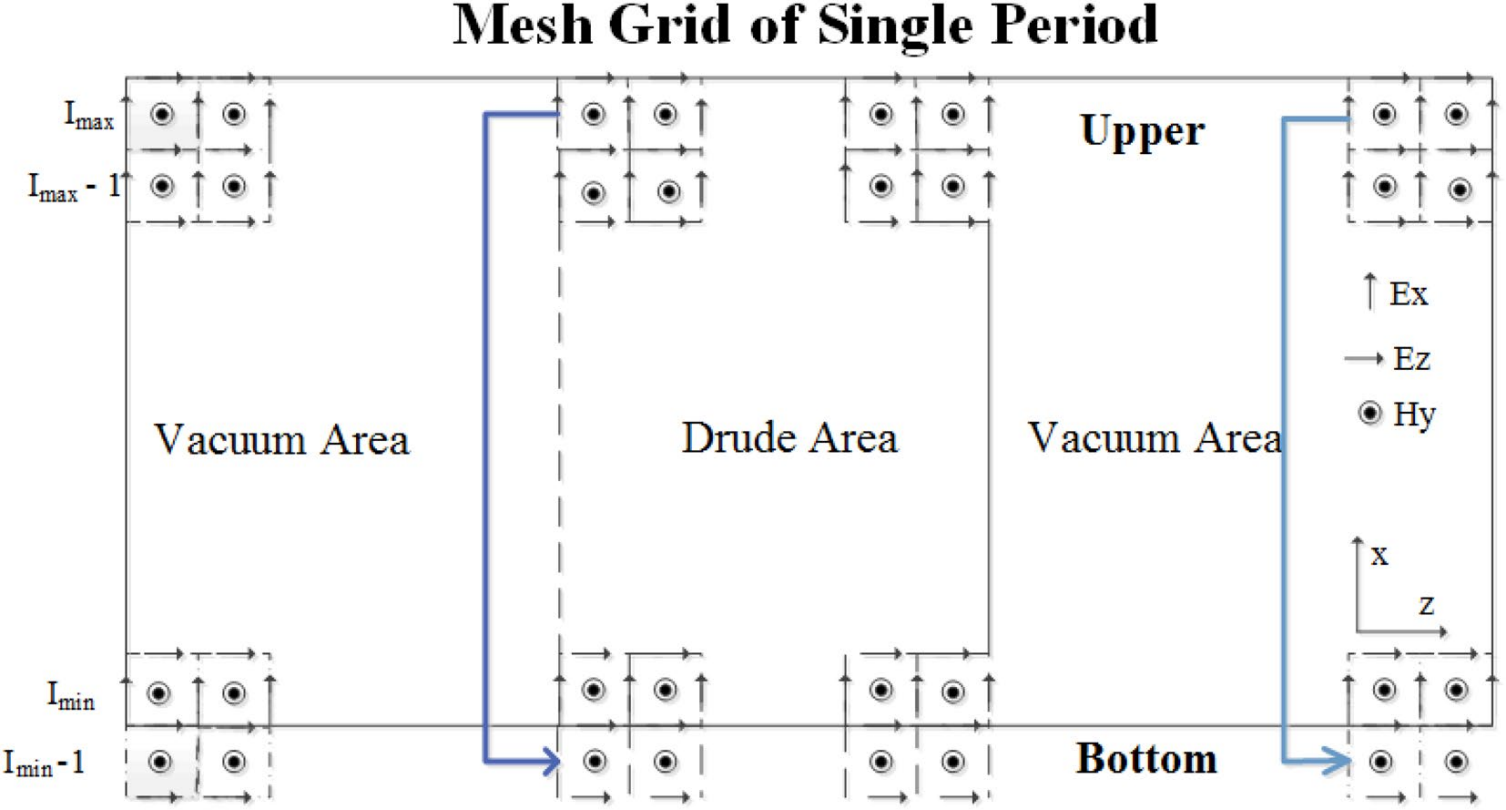
Electric and magnetic field in the mesh grid of a periodic structure.

To obtain the solution of Maxwell’s equations for the periodic structure, the PBC is introduced. Figure 1 shows the sketch picture of electric and the magnetic fields in a periodic structure. The PBC is used at upper and bottom of the x-direction. The boundary of z-direction in computational area is truncated by the proposed PML scheme. The boundary of the x-direction in the computational area is surrounded by PBCs due to its periodicity of the structure. As shown Fig. 1, when the PBC is added to the formula, we have the boundary conditions on the bottom given by

31 $$ \left. {H_{y}^{n + 1} } \right|_{{i_{\min } - 1/2}} = \left. {H_{y}^{n + 1} } \right|_{{i_{\max } - 1/2}} $$

32 $$ \left. {E_{z}^{n + 1} } \right|_{{i_{\min } }} = \left. {E_{z}^{n + 1} } \right|_{{i_{\max } }} $$

For the top boundary condition, we obtain

33 $$ \left. {H_{y}^{n + 1} } \right|_{{i_{\max } + 1/2}} = \left. {H_{y}^{n + 1} } \right|_{{i_{\min } + 1/2}} $$

34 $$ \left. {E_{z}^{n + 1} } \right|_{{i_{\min } }} = \left. {E_{z}^{n + 1} } \right|_{{i_{\max } }} $$

Thus, by employing the PBC, the matrices in (29)-(30) can be given as

35 $$ \left( {\begin{array}{*{20}c} {b_{i\_\min } } & {a_{i\_\min } } & {} & {} & {} & {} & {a_{i\_\min } } & {} \\ {a_{i\_\min + 1} } & {b_{i\_\min + 1} } & {a_{i\_\min + 1} } & {} & {} & {} & {} & {} \\ {} & \ddots & \ddots & \ddots & {} & {} & {} & {} \\ {} & {} & \ddots & \ddots & \ddots & {} & {} & {} \\ {} & {} & {} & \ddots & \ddots & \ddots & {} & {} \\ {} & {} & {} & {} & \ddots & \ddots & \ddots & {} \\ {} & {} & {} & {} & {} & {a_{i\_\max - 1} } & {b_{i\_\max } } & {a_{i\_\max - 1} } \\ {} & {a_{i\_\max } } & {} & {} & {} & {} & {a_{i\_\max } } & {b_{i\_\max } } \\ \end{array} } \right) $$

It can be observed that the matrices are no longer tri-diagonal matrices which can not be solve by employing the Thomas algorithm. Thus, an alternative method, the RCM method is employed to decrease the dimension of the matrices and improve the computational efficiency^27^.

## Numerical results and discussion

### Effectiveness of the proposed unconditionally stable implementation

**Figure 2:**
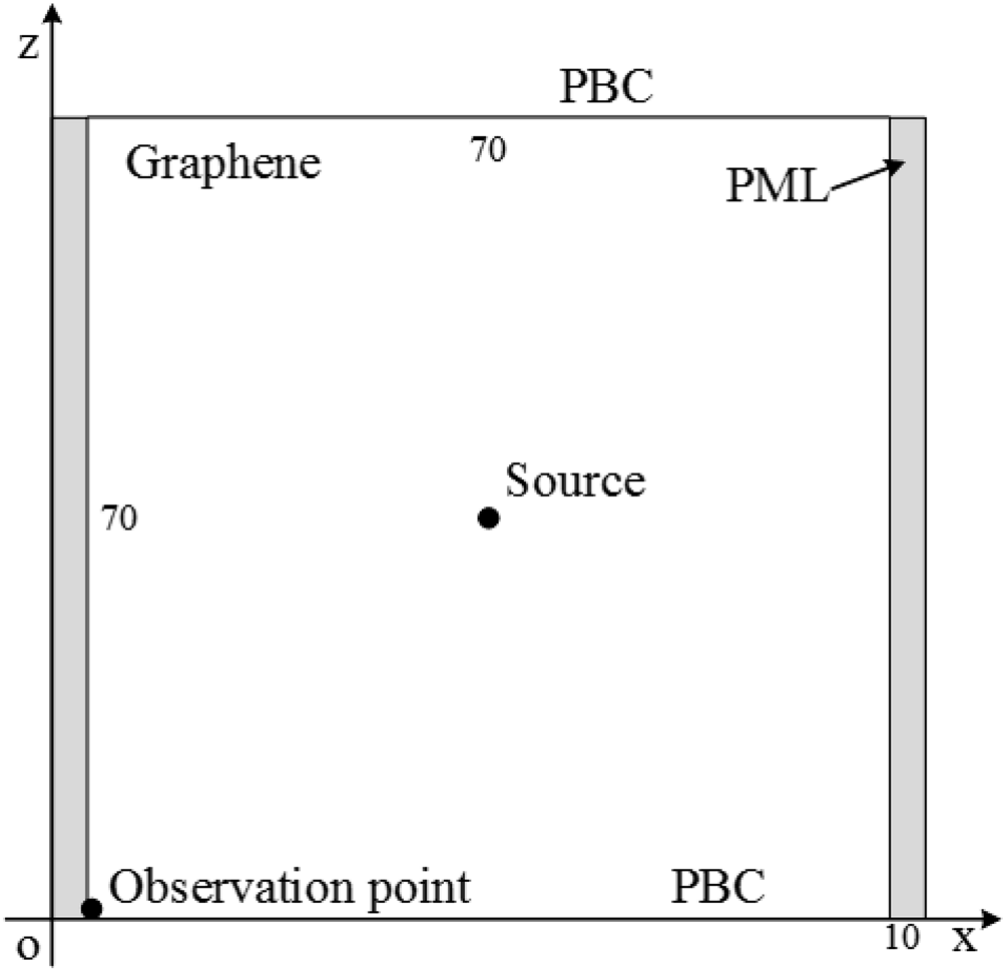
Sketch picture of the full-filled computational domain with graphene.

Before the investigation of GSs, the effectiveness and efficiency of the proposed CNDG-HO-PML algorithm is validated through a full-filled domain with graphene with the parameters of $T_{0} = 300\;K$, $\omega_{p} = 2.42 \times 10^{13} \;s^{ - 1}$, $\varepsilon_{ \propto } = 7$ and $\nu = 1.93 \times 10^{11} \;s^{ - 1}$, where $T_{0}$ is the environment temperature. The sketch picture of the computational domain is shown in Fig. 2. The computational domain has dimensions of ${70}\Delta x \times {70}\Delta z$. At the boundaries of the x-direction, 10-cell-PML is employed. At the upper and bottom of boundaries of z-direction, PBC is employed. The source which locates at the center of the domain is a modulated Gaussian pulse. The source can be expressed as

36 $$ J_{z} \left( t \right) = \sin \left[ {2\pi f_{c} \left( {t - t_{0} } \right)} \right]\exp \left[ { - \frac{{2\pi \left( {t - t_{0} } \right)^{2} }}{{\tau^{2} }}} \right] $$

where $\tau$ determines the pulse width, which can be obtained from the bandwidth that is 5000 THz_,_ $f_{c}$ is the center frequency. In this simulation, $f_{c} = 8500$ THz, $\tau = 0.2$, and $t_{0} = 4\tau$ are chosen^28^. The observation point is located at the left bottom corner of the domain to observe the waveform.

To ensure the accuracy of the calculation and validate the effectiveness of the algorithm, cell per wavelength (CPW) is chosen as 200. The CFL number (CFLN) is defined as $CFLN = {{\Delta t} \mathord{\left/ {\vphantom {{\Delta t} {\Delta t_{\max }^{FDTD} }}} \right. \kern-\nulldelimiterspace} {\Delta t_{\max }^{FDTD} }}$, where $\Delta t_{\max }^{FDTD}$ is the maximum time step of the conventional FDTD algorithm. The mesh size is $\Delta x = \Delta z = \Delta = 0.3$ nm. And the time step is $\Delta t_{\max }^{FDTD} = 7.07 \times 10^{ - 19}$ s. Inside the PML regions, the parameters are chosen to obtain the best performance both in the time domain and frequency domain. To make a comparison between the proposed scheme and previously works, the HO-PML based on the conventional FDTD algorithm (FDTD-HO-PML) is employed. Within the PML, the parameters are optimized to obtain the best absorbing performance.

**Figure 3:**
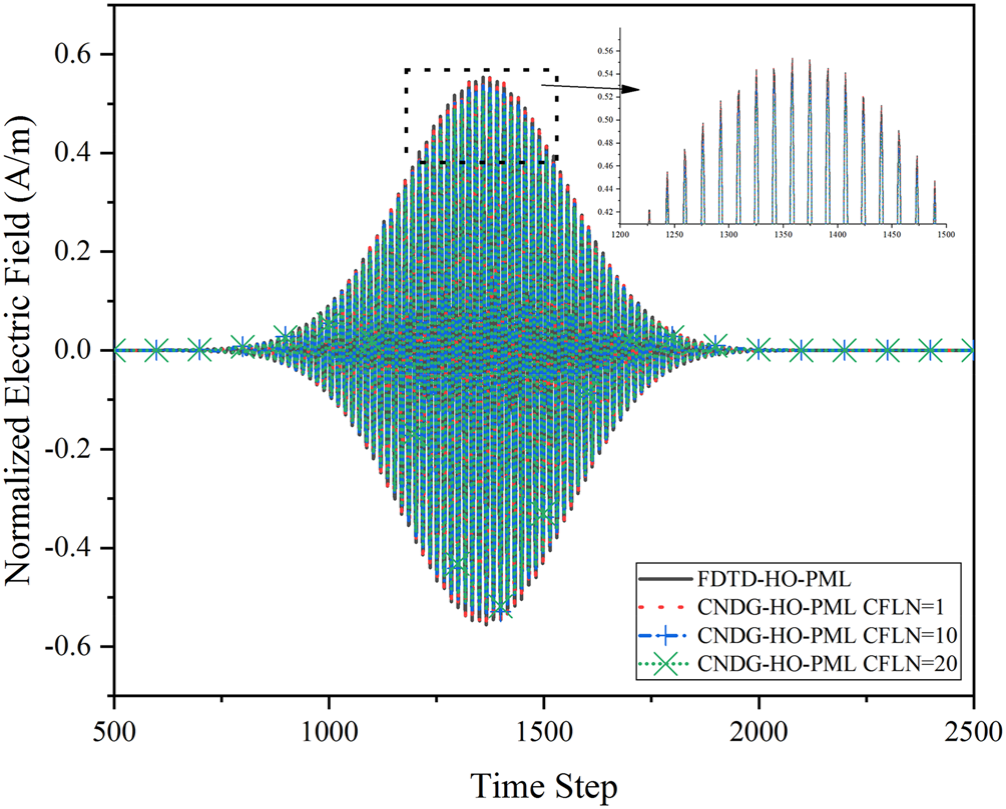
Waveform of the full-filled computational domain obtains from the observation point.

The waveform observed at the observation point obtained by different CFLNs are shown in Fig. 3. All of the waveforms are almost overlapped. In addition, it can be observed that although the computational accuracy degenerates with the increment of CFLNs, the proposed implementations when CFLN = 20 shows higher accuracy.

The accuracy of the calculation also affects by the absorbing performance of the PML regions. The absorbing performance of the PML regions can be reflected by the relative reflection error in the time domain which can be defined as

37 $$ R_{dB} \left( t \right) = 20\log_{10} \left[ {\left| {E_{x}^{t} \left( t \right) - E_{x}^{r} \left( t \right)} \right|/\left| {\max \{ E_{x}^{r} \left( t \right)\} } \right|} \right] $$

where $E_{x}^{t} \left( t \right)$ is the test solution which can be observed directly from the observation point and $E_{x}^{r} \left( t \right)$ is the reference solution which can be obtained by enlarging the computational domain to $7000\Delta x \times 7000\Delta z$ and terminating by 128-cell-PML without changing the relative position between the source and observation point. During the calculation of the reference solution, the reflection waves has no effected on the waveform.

**Figure 4:**
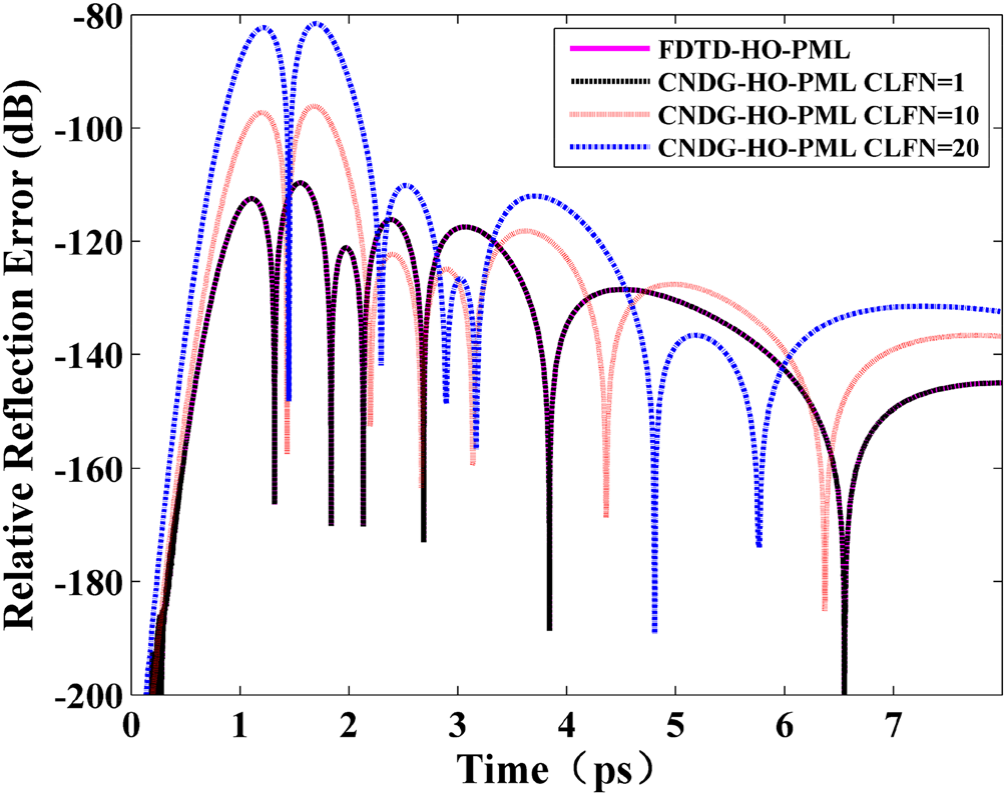
The relative reflection error versus time obtained by different algorithms and different CFLNs.

Figure 4 shows the relative reflection error obtained by different algorithms with different CFLNs. The absorbing performance degenerates with the increment of CFLNs. The maximum value of the relative reflection error of FDTD-HO-PML, CNDG-HO-PML CFLN = 1, 10 and 20 are − 109 dB, − 96 dB and − 81 dB, respectively. Although the absorbing performance decreases 21 dB when CFLN = 20, the proposed scheme can still be employed in the practical engineering (usually below − 40 dB)^29^. The computational efficiency, memory consumption and time reduction are shown in Table 1. As is shown that computational efficiency decreases when CFLN = 1. The reason is that the two matrices obtained by RCM method must be calculated at each time step resulting in such condition. The computational efficiency can be further improved by employing larger CFLNs to reduce the iteration steps. It can be observed that when CFLN = 10 and 20, the CPU time reduces by 75.0% and 87.3%, respectively. This indicating the computational efficiency can be obviously improved by employing the proposed scheme. In conclusion, the proposed scheme shows considerable performance and accuracy. Thus, the following investigations are based on CNDG-HO-PML when CFLN = 20. The FDTD-HO-PML chosen for comparison.

**Table 1:** The computational efficiency, memory consumption and time reduction with different PML implementations and CFLNs.

PML algorithm	Memory (MB)	Time (s)	Time reduction (%)
FDTD-CFS-PML	3.0	67.6	–
2ND-CNAD-CFS-PML (CFLN = 1)	3.4	148.5	− 119.7
2ND-CNAD-CFS-PML (CFLN = 10)	3.4	16.9	75.0
2ND-CNAD-CFS-PML (CFLN = 20)	3.4	8.6	87.3

### Wave transmission through graphene sheet

The wave transmission through a graphene sheet is studied with regard to transverse electric $\left( {TE_{y} } \right)$ mode. The graphene sheet is composed of single-layer graphene with the thickness of 3.4 nm. The parameters of graphene are chosen as $T_{0} = 300\;K$, $\omega_{p} = 2.42 \times 10^{13} \;s^{ - 1}$, $\varepsilon_{ \propto } = 7$ and $\nu = 1.93 \times 10^{11} \;s^{ - 1}$.

**Figure 5:**
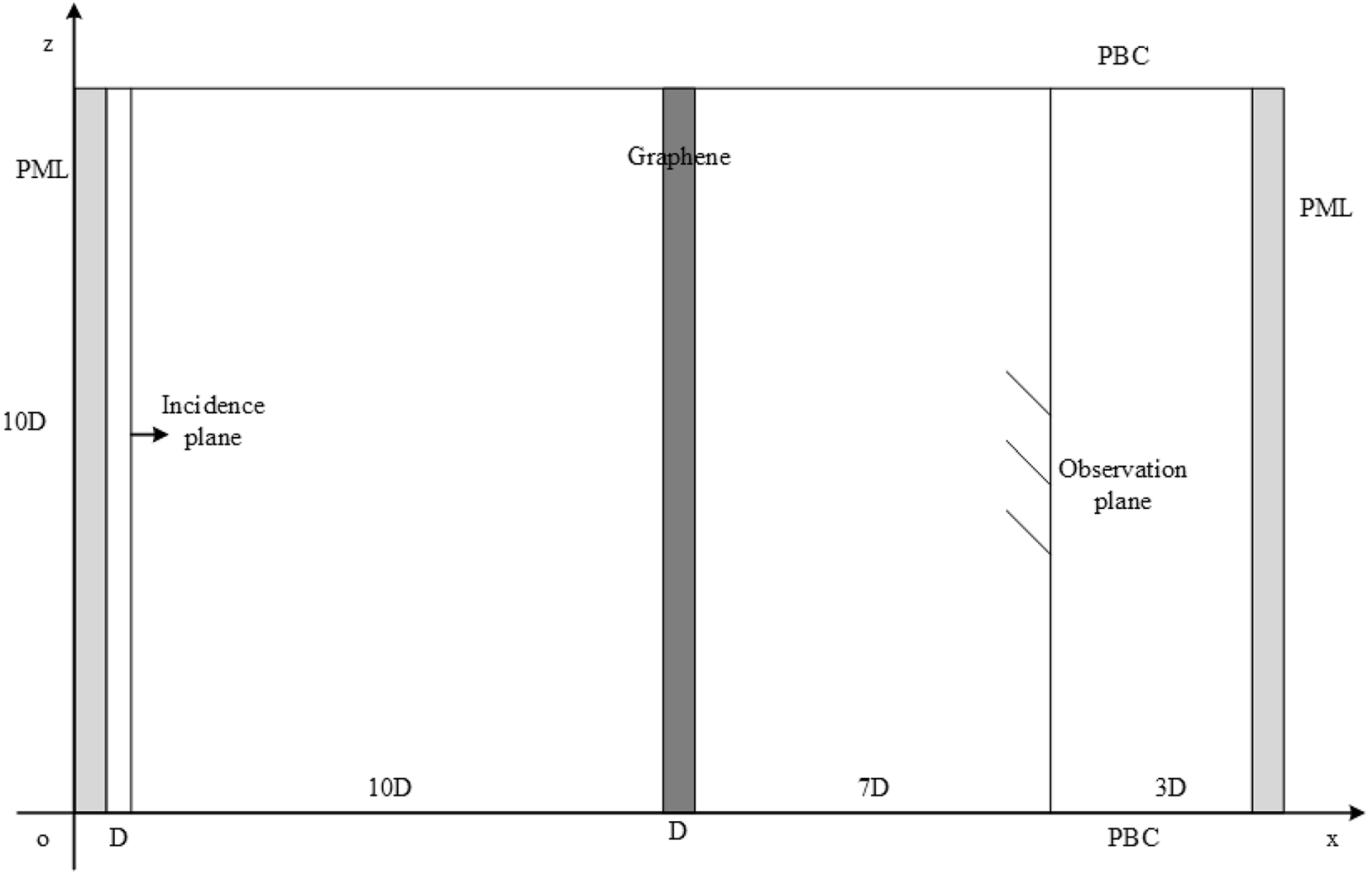
The sketch picture of the computational domain for wave transmission through graphene sheet.

The sketch picture of the computational domain for investigation is shown in Fig. 5. Assuming that the graphene has the thickness of $D$ with the unit of cells. The graphene is located at the center of the x-direction, the rest of the structure is filled with vacuum. The size of the computational domain is $21D \times 10D$. There is an x-polarized plane normally incident from vacuum into a graphene sheet along the positive direction of x-axis. The distance between the source and the PML regions is $D$. The source is a modulated Gaussian pulse with the bandwidth and center frequency of 5000 THz. The observation plane is located at the left side of PML regions with the distance of $3D$. The thickness of the GSs include 0.34 nm, 3.4 nm ($D$ = 1, 10 cells), 6.8 nm ($D$ = 2, 20 cells), 13.6 nm ($D$ = 4, 40 cells) 20.4 nm ($D$ = 6, 60 cells) and 27.2 nm ($D$ = 8, 80 cells). The boundaries of z-direction in the computational domain are truncated by PBC due to its periodicity of the structure PML. The boundaries of x-direction in the computational domain are terminated by 10-cell-PML.

It should be noticed that the mesh size and the time step is no longer limited by the CFL condition. Thus, the physical size can be employed during the simulation. To ensure the accuracy of calculation, the thickness of single-layer GS is divided into 10 cells as $\Delta x = \Delta z = \Delta = 0.34$ nm. The time step can be obtained from $\Delta t = {\Delta \mathord{\left/ {\vphantom {\Delta {\sqrt 2 }}} \right. \kern-\nulldelimiterspace} {\sqrt 2 }}c$ as $8.02 \times 10^{ - 19}$ s.

**Figure 6:**
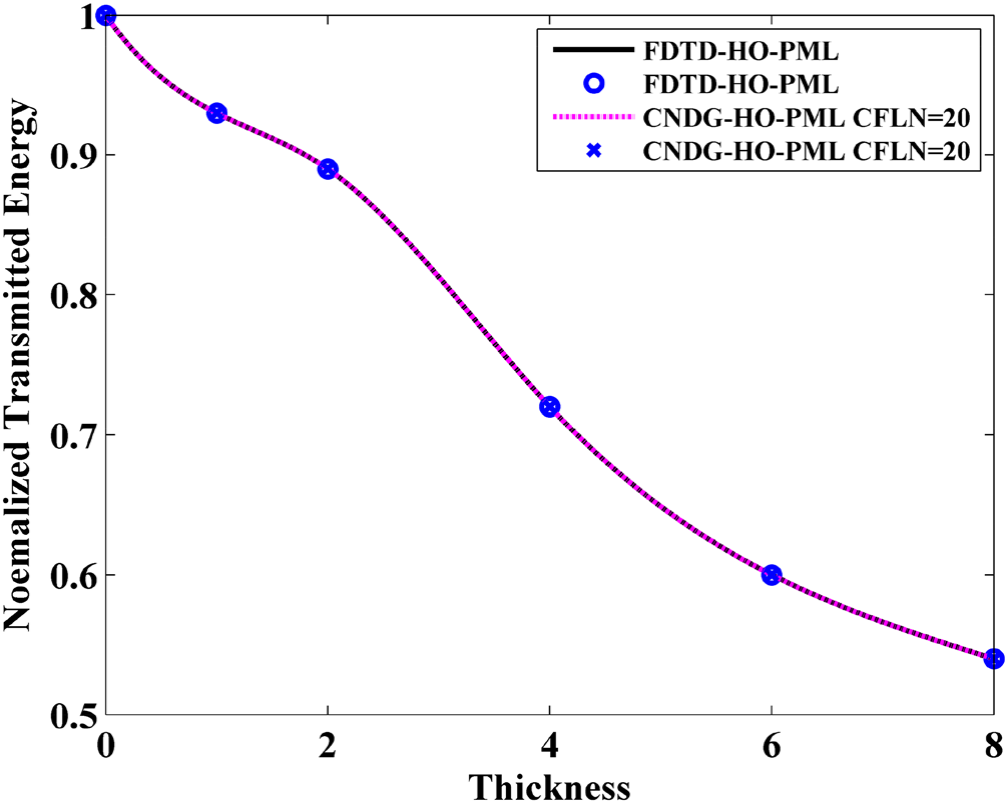
Normalized transmitted energy versus different thickness obtained by FDTD-HO-PML and CNDG-HO-PML CFLN = 20.

Firstly, the wave transmission through GSs can be investigated through the maximum value of the waveform obtained from the observation point. Figure 6 shows the normalized transmitted energy versus different thickness obtained by FDTD-HO-PML and CNDG-HO-PML CFLN = 20. It can be observed that the curves are almost overlapped indicating they hold same accuracy. The CPU time of FDTD-HO-PML and CNDG-HO-PML CFLN = 20 are 78.3 s and 10.6 s, respectively. By employing the proposed scheme, the CPU time can be decreased by 86.5%. Thus, the effectiveness and the accuracy of the proposed scheme have been demonstrated.

**Figure 7:**
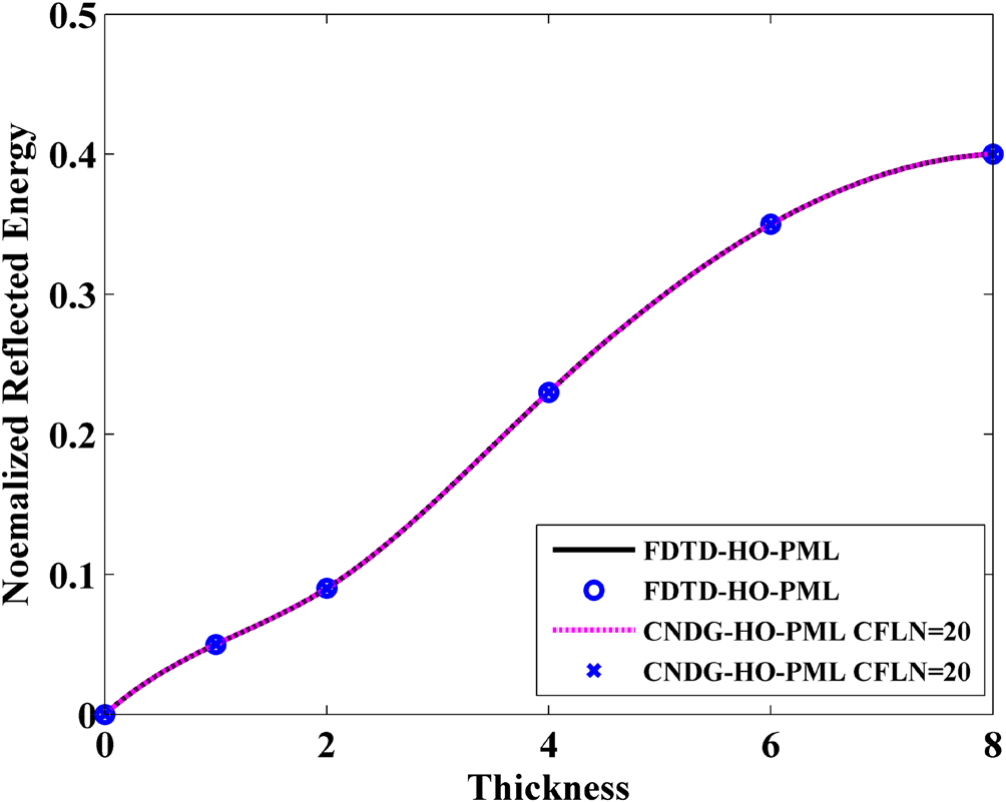
Normalized reflected energy versus different thickness obtained by FDTD-HO-PML and CNDG-HO-PML CFLN = 20.

**Figure 8:**
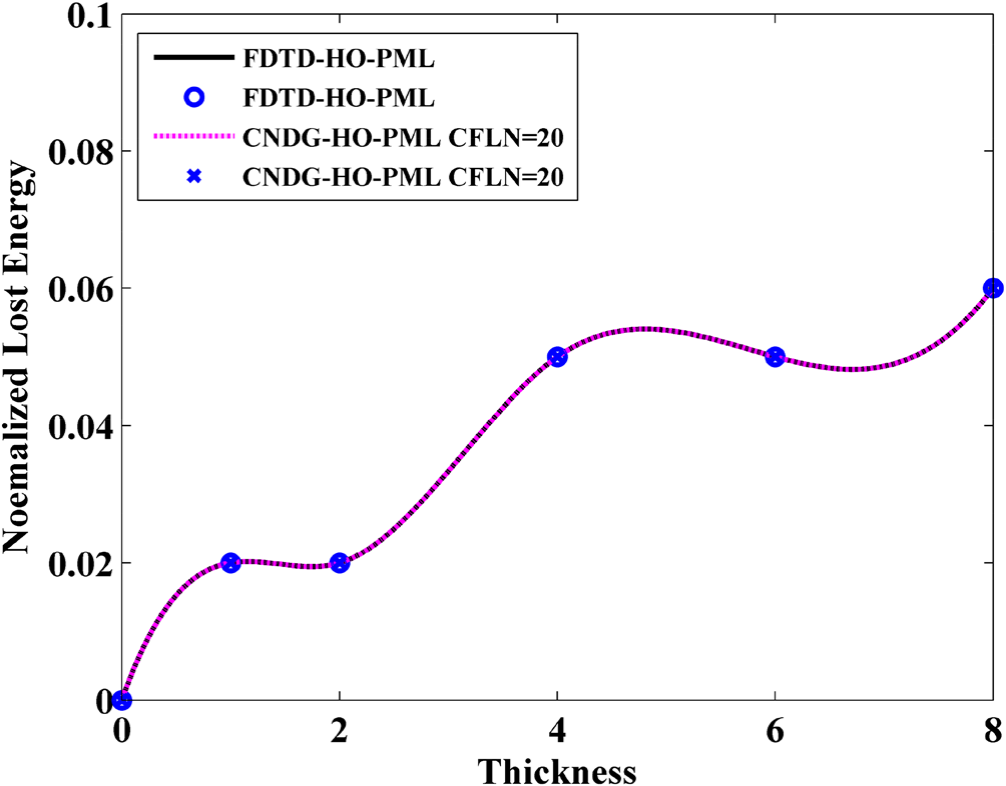
Normalized lost energy versus different thickness obtained by FDTD-HO-PML and CNDG-HO-PML CFLN = 20.

In addition, it can be observed that the normalized transmitted energy decreases with the increment of the thickness. This indicates that the thicker sheet transmits less energy. This phenomenon can be reflected by the normalized reflection energy, shown in Fig. 7. It can be observed that the reflection energy increases with the increment of thickness. Especially, it can be founded that by adding the normalized reflection energy and the normalized transmitted energy, the resultant is no longer 1 at some cases. This indicates that a part of energy is lost by the sheet. The normalized loss energy is shown in Fig. 8. The results prove that thicker sheets losses much energy during the wave transmission.

**Figure 9:**
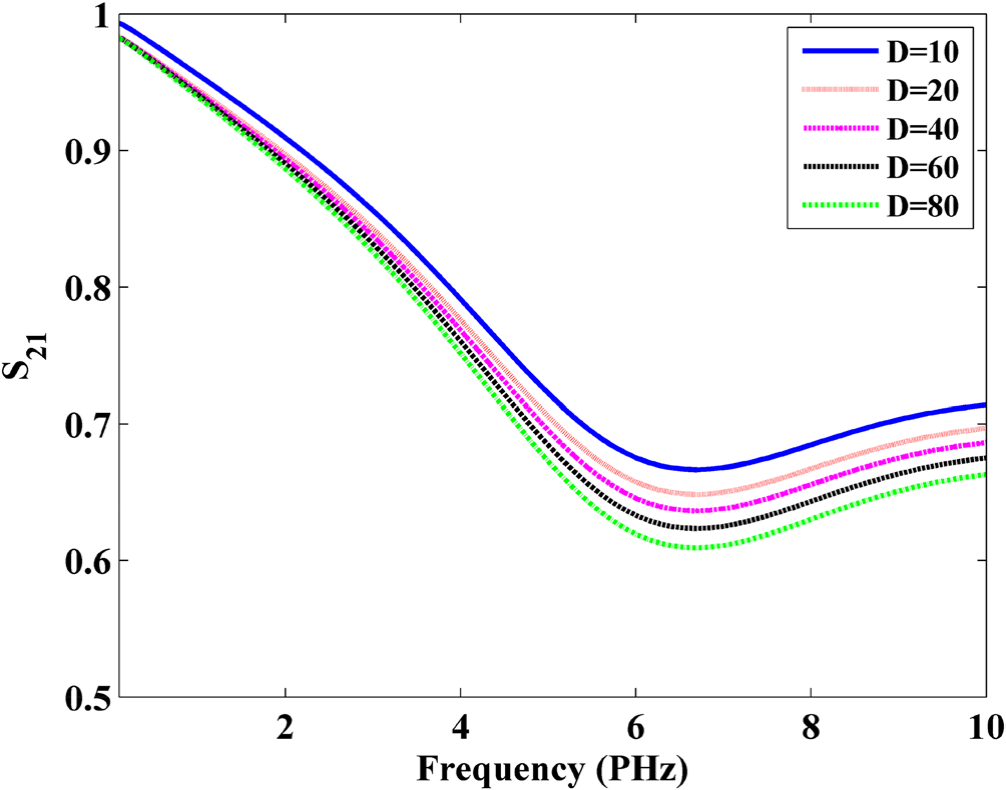
Frequency versus S21 parameters obtained by different thickness.

**Figure 10:**
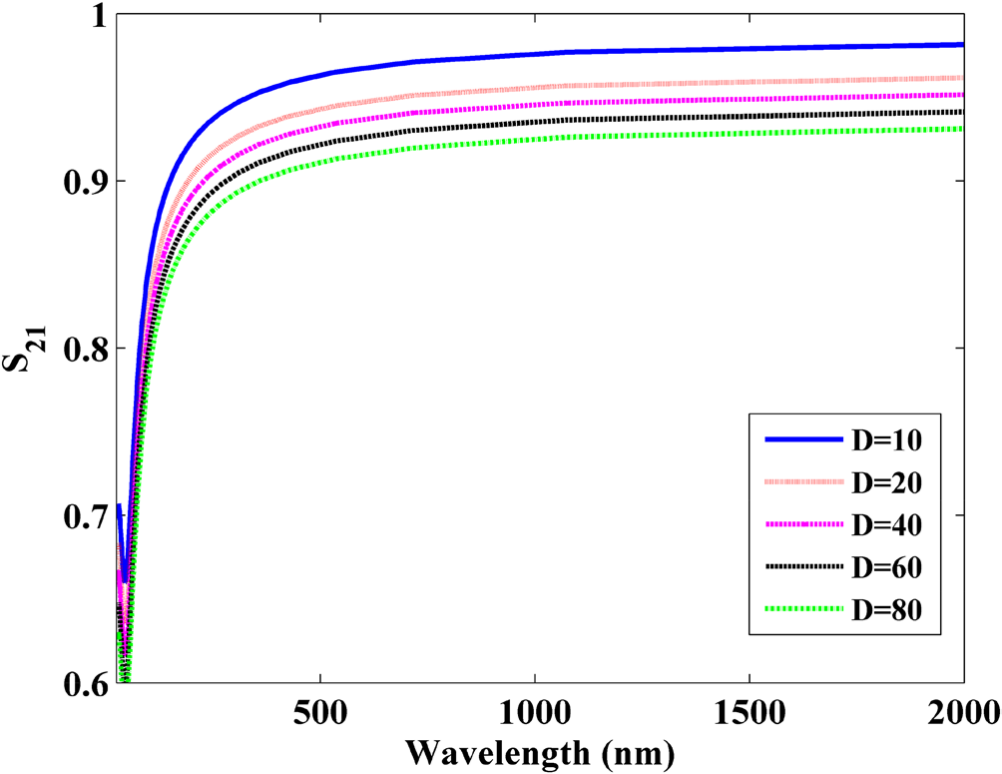
Wavelength versus S21 parameters obtained by different thickness.

To demonstrate the wave transmission in the frequency domain, the S parameters in the microwave theory is introduced during the simulation^30^. As defined in the microwave theory, the transmission can be reflected by S_21_. Thus, the port 1 is defined at the left side of sheet with the incidence plane wave. Port 2 is defined at the right side of the sheet. In this simulation, the S_21_ is obtained by CNDG-HO-PML when CFLN = 20, as shown in Fig. 9. It can be observed that S_21_ decreases with the increment of the thickness. At the frequency of 6.2 PHz, the graphene sheets show the least S parameters. At the low-frequency, the graphene sheets have higher S parameters indicating the less loss during the wave transmission. Figure 10 show the wavelength versus S_21_ parameters obtained by different thickness. It can be observed that the S_21_ becomes lower in small wavelength.

### Extraordinary optical transmission through graphene sheet

**Figure 11:**
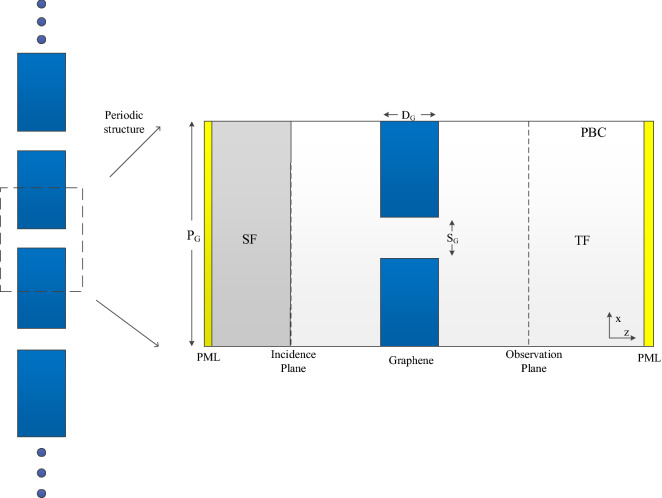
The sketch picture of periodic graphene structure with slit and its single unit.

To investigate the extraordinary optical transmission phenomenon through the graphene sheet, a periodic graphene structure with slit is proposed. Figure 11 demonstrates the constitution of the whole structure and its single unit.

$P_{G}$, $D_{G}$, $S_{G}$ represent the length of a single unit in the z-direction, the width of graphene sheet in x-direction and the length of slit in the z-direction, respectively. The whole computational domain of single unit is divided into two regions including the total field (TF) and scattering field (SF) regions. The TF region is located on the right side of the incidence plane and the SF is on the left of the incidence plane. The length of the SF in the z-direction is 10 cells. The distance between incidence plane and sheet is 50 cells. The distance between observation plane and GS is 50 cells as well. $P_{G}$ is 100 cells in this simulation. The width of the graphene $S_{G}$ changes from 6 to 36 cells. The thickness of the graphene $D_{G}$ changes from 10 to 80 cells. The excitation source, mesh size and time step are the same as the numerical example above. The resultants are obtained by CNDG-HO-PML with CFLN = 20.

**Figure 12:**
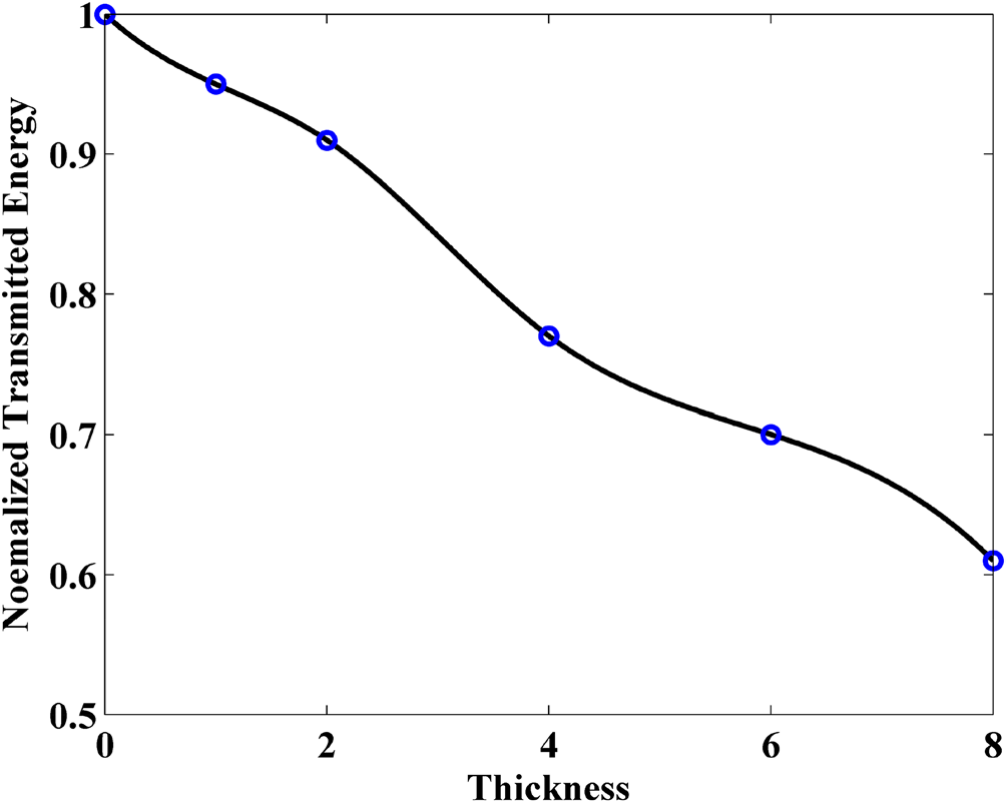
Normalized transmitted energy versus different thickness obtained by CNDG-HO-PML CFLN = 20.

**Figure 13:**
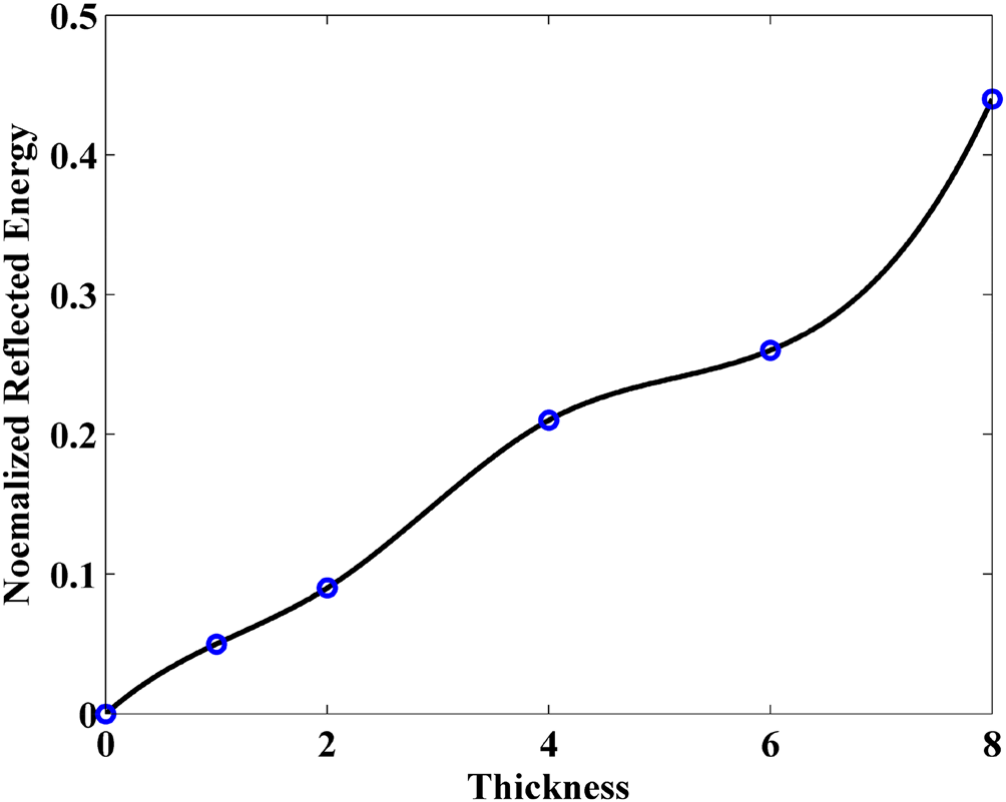
Normalized reflected energy versus different thickness obtained by CNDG-HO-PML CFLN = 20.

**Figure 14:**
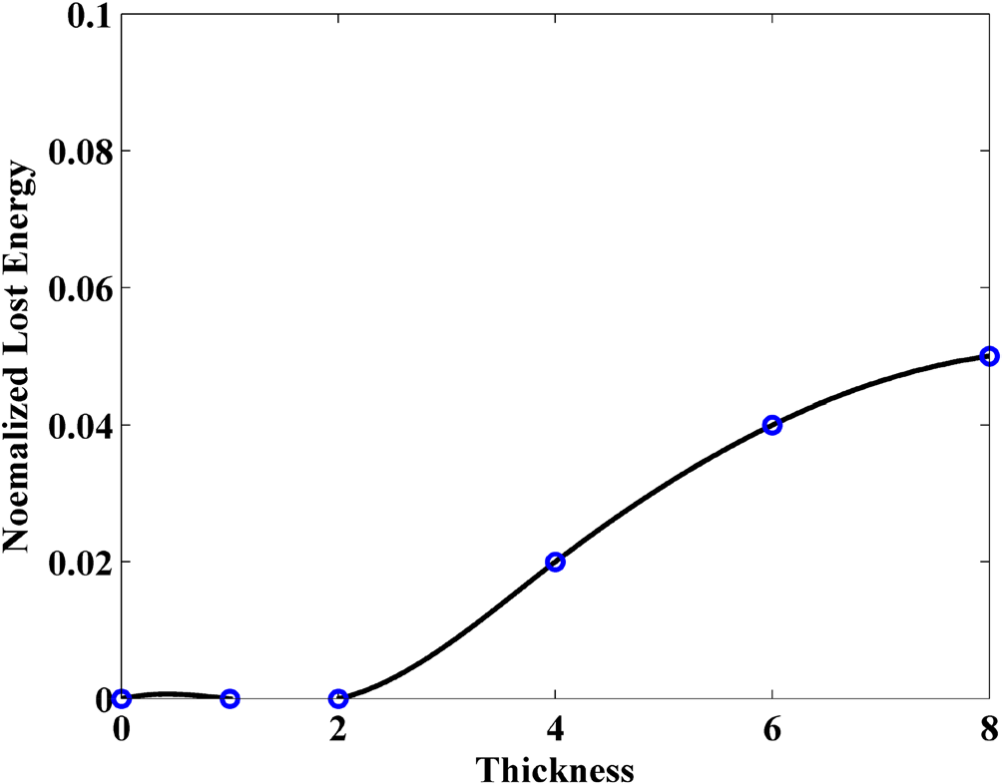
Normalized lost energy versus different thickness obtained by CNDG-HO-PML CFLN = 20.

**Figure 15:**
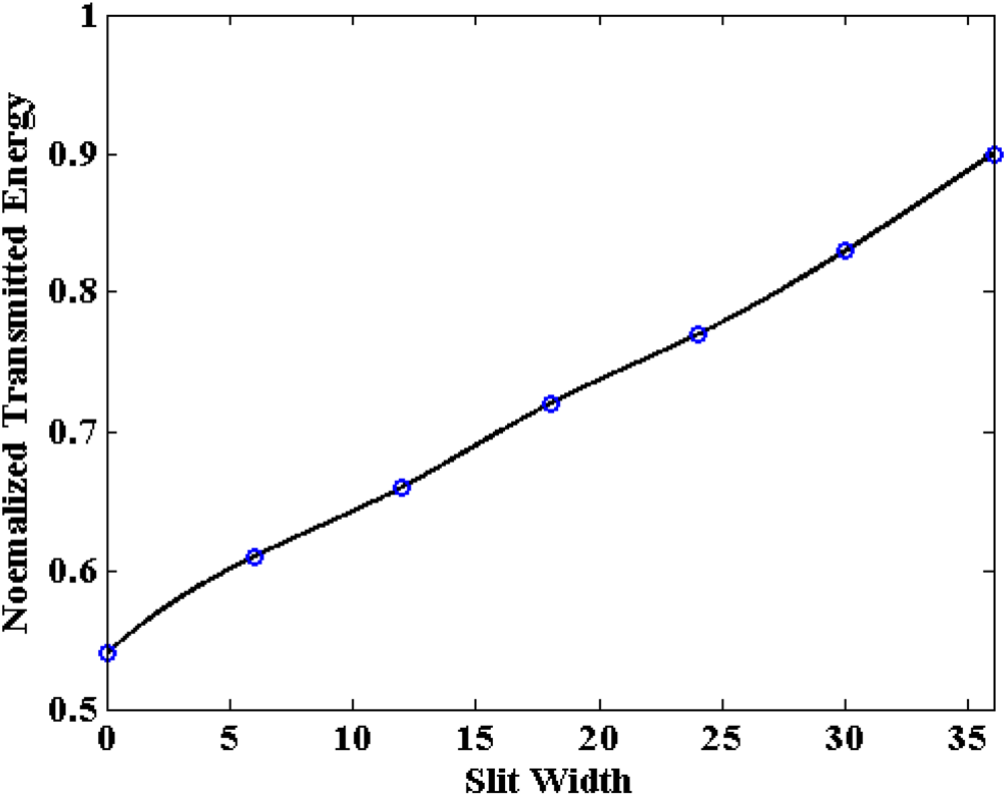
Normalized transmitted energy versus different slit width obtained by CNDG-HO-PML CFLN = 20.

**Figure 16:**
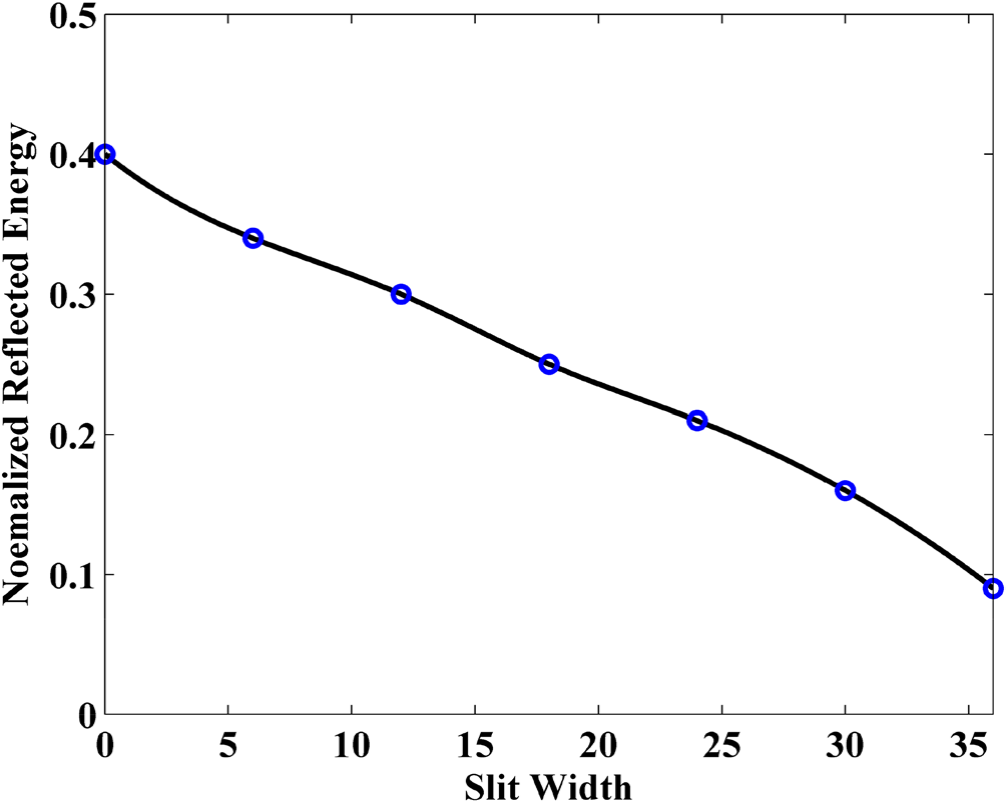
Normalized reflected energy versus different slit width obtained by CNDG-HO-PML CFLN = 20.

**Figure 17:**
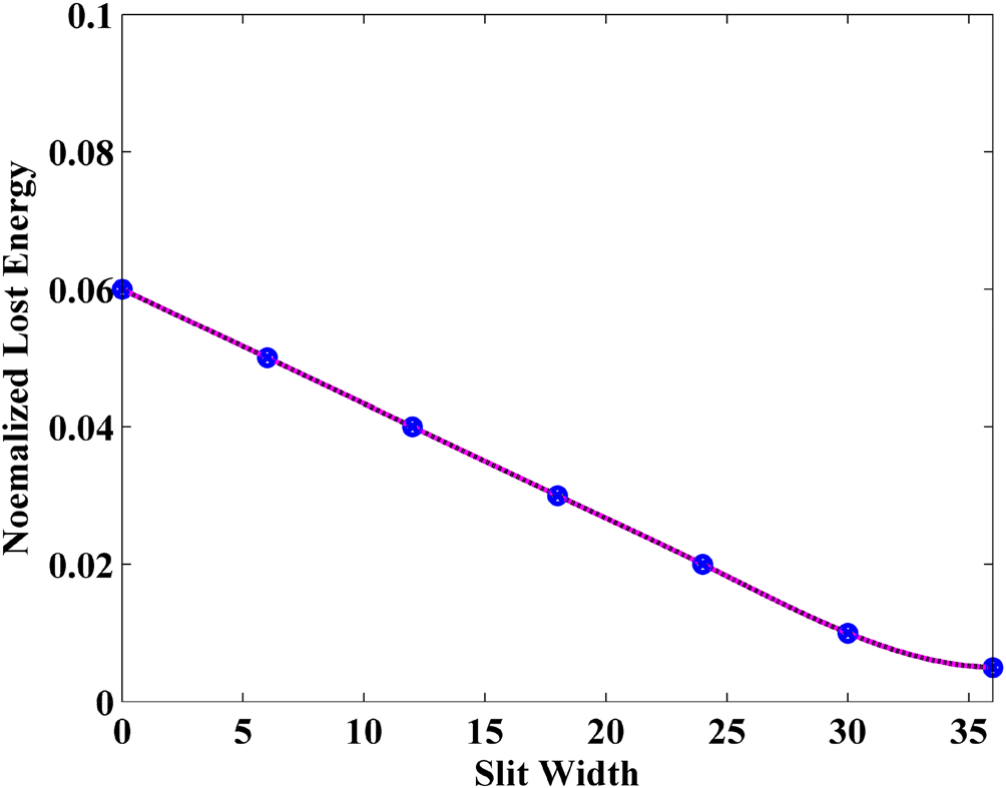
Normalized lost energy versus different slit width obtained by CNDG-HO-PML CFLN = 20.

Compared with the graphene sheet without slit in the previously numerical example, due to the existence of slit, the interference of wave transmission results in the generation of the crest and trough in the observation plane. Thus, it should be noticed that the energy from each mesh grid in the observation plane should be employed during the calculation. Figures 12, 13 and 14 show the normalized transmitted energy, normalized reflected energy, and normalized lost energy versus different thickness with the slit width of 6 cells. It can be observed that the transmitted energy decreases with the increment of thickness. The normalized reflected energy increases at the same time. Meanwhile, compared with the normalized lost energy in the graphene sheet, it decreases in this numerical example. The reason is that due to the existence of the slit, less energy is consumed during the wave transmission of the graphene. In addition, compared with the single graphene sheet, the normalized transmitted energy through the graphene grating becomes larger. The electric current that pass through the slit causes such phenomenon. Figures 15, 16 and 17 show the normalized transmitted energy, normalized reflected energy, and normalized lost energy versus different slit width with the 8 layers thick sheet. It can be observed that the transmitted energy increases with the increment of the slit width. The reason is that large slit width can transmit much power compared with the narrow slit. Meanwhile, the consumption energy of graphene decreases with the increment of width slit indicating that less energy is cost during the wave transmission.

**Figure 18:**
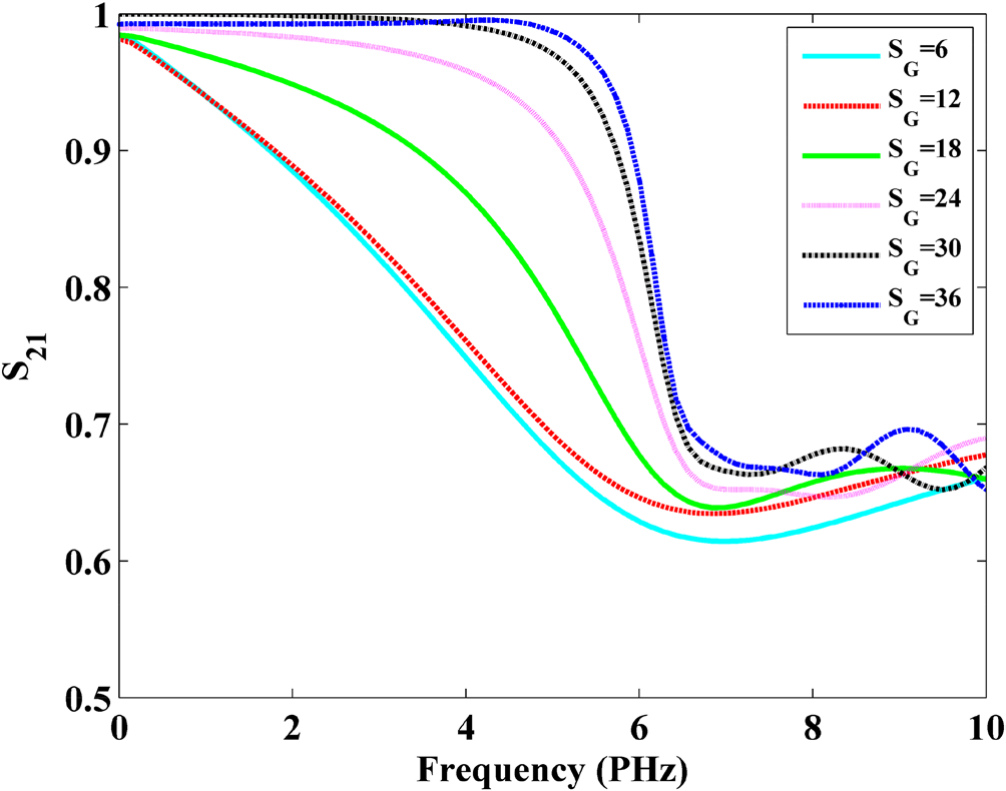
Frequency versus S21 parameters obtained by slit width.

**Figure 19:**
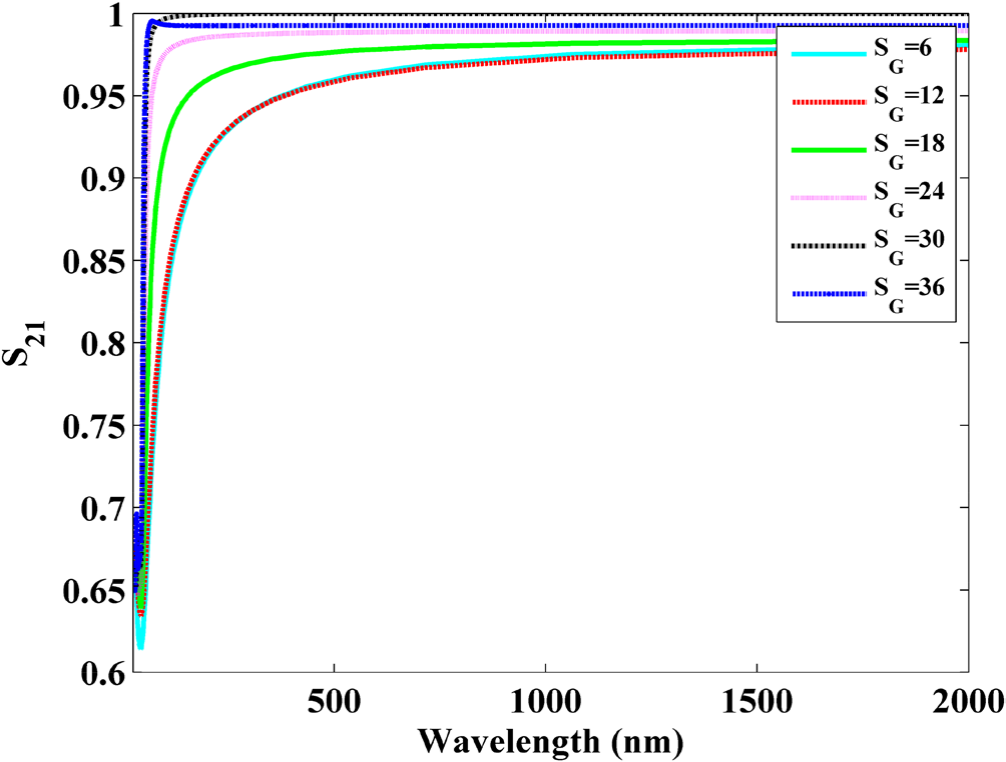
Wavelength versus S21 parameters obtained by slit width.

Following the same step as the numerical example above, the S_21_ parameters can be employed in evaluating the performance and investigating the properties of graphene. Figures 18 and 19 show the S_21_ parameters versus frequency and wavelength with different slit width under 8-layer-sheet, respectively. It can be observed that the S parameters increases obviously with the increment of slit width. This indicates that the wave is much easier to transmit with the increment of slit width before the 6 PHz frequency.

As is demonstrate in the previously that the wave is no longer uniform at the observation plane. The reason is that the interference of electric current occurs when wave transmitting through the slit. The crest and the trough are formed at the observation point. Thus, it is important to investigate the crest and the trough of the waveform.

In this simulation, the parameter $\eta = {{\left| {\max \left\{ {H_{t} \left( t \right)} \right\}} \right|^{2} } \mathord{\left/ {\vphantom {{\left| {\max \left\{ {H_{t} \left( t \right)} \right\}} \right|^{2} } {\left| {\max \left\{ {H_{r} \left( t \right)} \right\}} \right|^{2} }}} \right. \kern-\nulldelimiterspace} {\left| {\max \left\{ {H_{r} \left( t \right)} \right\}} \right|^{2} }}$ is defined as the ratio of the field intensity at the observation plane, where $H_{t} \left( t \right)$ is the magnetic field different situations, $H_{r} \left( t \right)$ is the magnetic field under the circumstance of single sheet^31–34^.

**Figure 20:**
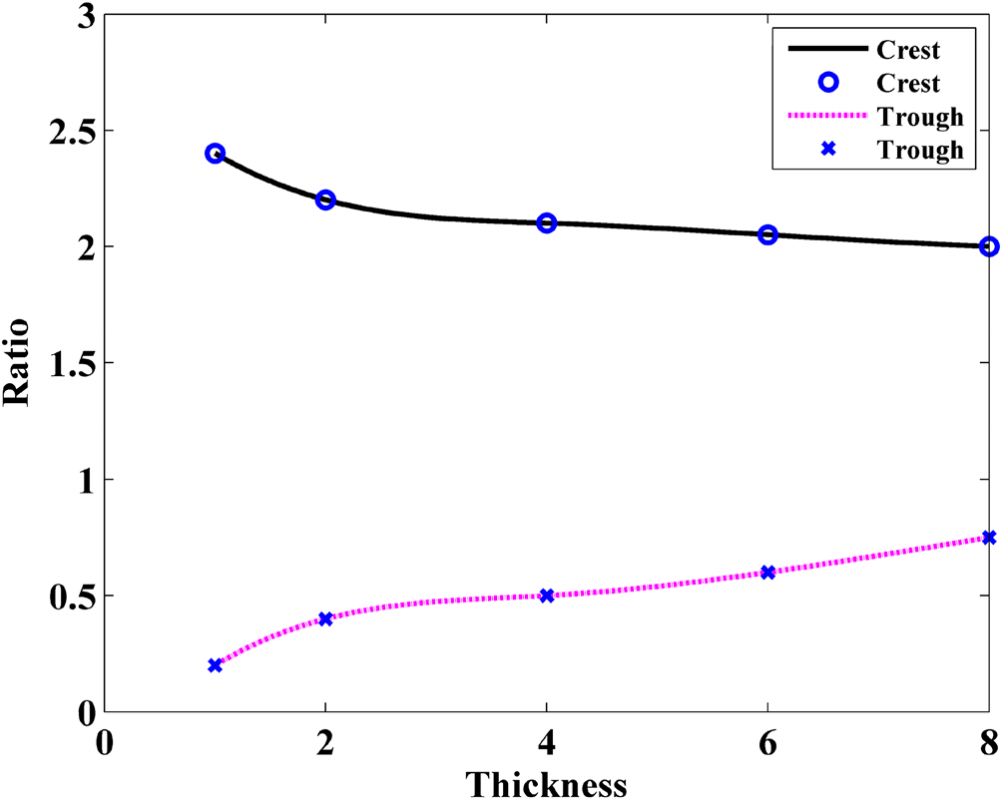
Relationship between the ratio of the field intensity and the layer thickness.

**Figure 21:**
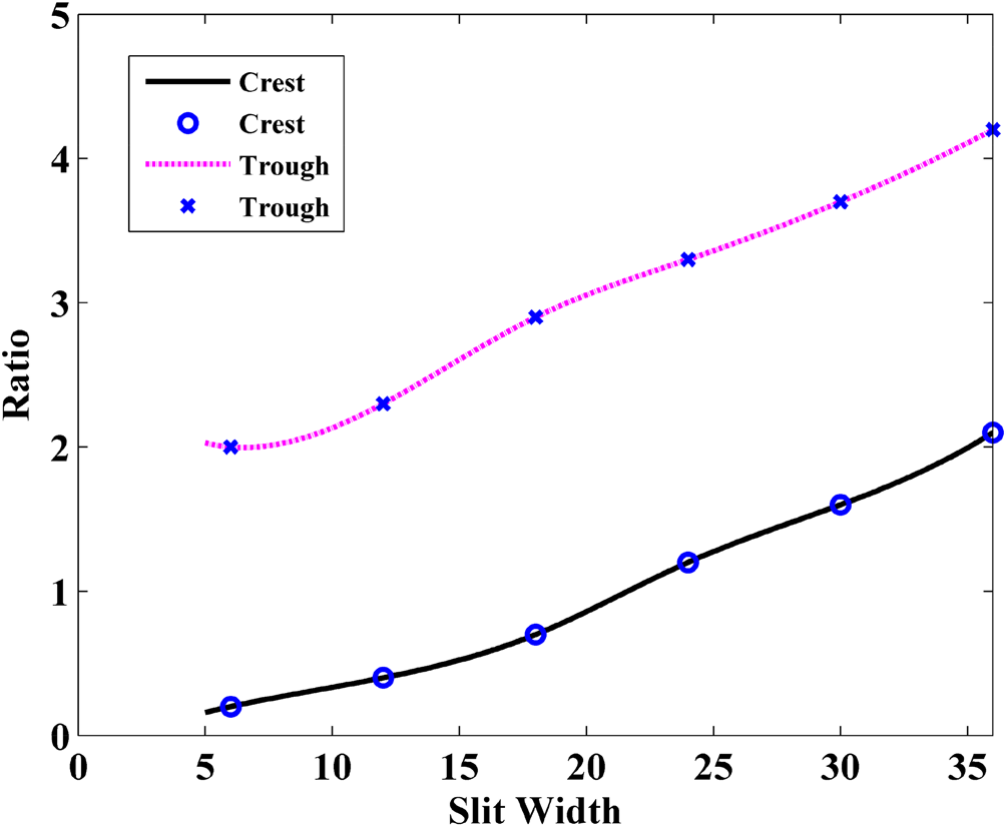
Relationship between the ratio of the field intensity and the slit width.

Figures 20 and 21 demonstrate the ratio versus thickness and silt width, respectively. As is shown in Fig. 20 that the influences of graphene gratings on the magnitude are significantly placid when the graphene thickness is larger than 4 layers. As shown in Fig. 21, the ratio of the wave intensity increases linearly with slit widths. This can be explained that when the thickness of the GS increases, part of the electric current pass through the slit. The portion which traverse the GS suffers more loss. Therefore, the effect of wave interference that causes by the electric current becomes weak. In conclusion, the ratio between crest and trough is coincidence not only with the trend of energy but also with the S_21_ parameters.

## Conclusions

Based on the CNDG algorithm, the Drude-like model and higher order concept, an unconditionally stable CNDG-HO-PML algorithm is presented to investigate the wave propagation through graphene gratings. The Maxwell’s equations are applied to the model to study the transmission characteristics. The ADE- and PLRC-FDTD methods with faster calculation and less memory is used to solve the partial differential equations for time stepping of the electromagnetic fields. The effectiveness and efficiency are testified through a full-filled structure, a single sheet and the sheet with slit. Based on numerical analysis, the obtained result shows that the characteristics of graphene with high transmission performance. The grating graphene layer structures can be used in the field of optoelectronic detection technology and systems.
